# Multimodal *in vivo* imaging reveals limited allograft survival, intrapulmonary cell trapping and minimal evidence for ischemia-directed BMSC homing

**DOI:** 10.1186/1472-6750-12-93

**Published:** 2012-12-03

**Authors:** Bert R Everaert, Irene Bergwerf, Nathalie De Vocht, Peter Ponsaerts, Annemie Van Der Linden, Jean-Pierre Timmermans, Christiaan J Vrints

**Affiliations:** 1 Laboratory of Cell Biology and Histology, University of Antwerp, Antwerp, Belgium; 2 Laboratory of Cellular and Molecular Cardiology, Antwerp University Hospital, Antwerp, Belgium; 3 Laboratory of Experimental Hematology, Vaccine and Infectious Disease Institute (Vaxinfectio), University of Antwerp, Antwerp, Belgium; 4 BioImaging Laboratory, University of Antwerp, Antwerp, Belgium; 5 Laboratory of Cell Biology and Histology, Groenenborgerlaan 171, Antwerp, 2020, Belgium

**Keywords:** Stem cell, BMSC, Homing, Bioluminescence, Confocal endomicroscopy

## Abstract

**Background:**

Despite positive reports on the efficacy of stem cell therapy for the treatment of cardiovascular disease, the nature of stem cell homing to ischemic tissues remains elusive.

**Results:**

We used a mouse model of peripheral tissue ischemia to study the survival and homing capacity of dual reporter gene (eGFP/Luciferase) expressing bone marrow-derived stromal cells (BMSC). Cell homing and survival were studied in the presence and absence of ciclosporin A (CsA) immunosuppression using bioluminescence imaging (BLI) together with confocal endomicroscopy. Different injection strategies were applied: central venous (CV), intra-arterial (IA) and intramuscular (IM). BLI and confocal endomicroscopy evidenced complete rejection of the IM injected allogeneic BMSC transplant within 5 to 10 days. Immunosuppression with CsA could only marginally prolong graft survival. IM injected BMSC did not migrate to the site of the arterial ligation. CV injection of BMSC resulted in massive pulmonary infarction, leading to respiratory failure and death. Intrapulmonary cell trapping was evidenced by confocal endomicroscopy, BLI and fluorescence microscopy. IA injection of BMSC proved to be a feasible and safe strategy to bypass the lung circulation. During the follow-up period, neither BLI nor confocal endomicroscopy revealed any convincing ischemia-directed homing of BMSC.

**Conclusions:**

BLI and confocal endomicroscopy are complementary imaging techniques for studying the in vivo biology of dual reporter gene-expressing BMSC. Allogeneic BMSC survival is limited in an immunocompetent host and cannot be preserved by CsA immunosuppression alone. We did not find substantial evidence for ischemia-directed BMSC homing and caution against CV injection of BMSC, which can lead to massive pulmonary infarction.

## Background

Mesenchymal stem cell (MSC) therapy is currently being explored in almost any clinical field involving tissue restoration or modulation of immune responses [1]. This widely applicable, simple and straightforward therapy would represent a milestone in modern medicine. The initial enthusiasm about the promising results of MSC therapy in preclinical animal studies, and later on also in phase 1 and phase 2 clinical trials was subsequently tempered by the outcome of larger randomized, placebo-controlled trials. The reasons herefore are not completely understood and probably relate to our lack of knowledge and understanding of the complex nature of MSC biology.

MSC were first described as clonogenic fibroblast precursor cells, residing in mouse bone marrow, thymus and spleen [2]. After 30 years of MSC research, the International Society for Cellular Therapy, in an effort to facilitate data exchange between investigators, came up with a set of minimal criteria to define human MSC. These cells should be plastic-adherent when maintained in standard culture conditions, have multilineage differentiation potential into osteoblasts, adipocytes and chondroblasts *in vitro* and express a specific set of cellular markers.

Despite the consensus on *in vitro* MSC characteristics, many unresolved questions remain about MSC biology *in vivo*, precluding its potential clinical applicability on a large scale [1,3]. Key questions on whether MSC truly undergo lineage differentiation and on the mechanisms through which these cells act on target tissues, i.e., their anti-apoptotic, pro-angiogenic or immune modulatory properties, are still matter of intense debate. Although many animal studies reported the positive effects of MSC therapy, usually very little information is provided about the long-term survival and engraftment of the administered MSC. A plausible explanation for this lack of information might be the absence of an easily applicable “gold standard” for studying cell survival and homing *in vivo*. Indeed, whole-body imaging techniques using radioactive or magnetic labeling dyes, fluorescent markers or reporter genes, all have disadvantages and probably a combination of different imaging modalities is needed to provide a reliable picture.

In addition, uncertainty remains about the optimal transplantation route, time frame and cell dose, and whether homing after systemic infusion is an active rather than passive “cell entrapment” phenomenon [4,5]. Finally, the safety of injecting vast amounts of foreign cells is a matter of concern, and although a relatively large amount of safety data has been gathered from phase 1 and 2 clinical trials, the observation that many systemically infused MSC become entrapped in the lungs warrants further investigation [6].

In the present study we combined two complementary *in vivo* imaging techniques, i.e., bioluminescence imaging (BLI) and confocal endomicroscopy, to study dual reporter gene-expressing stem cell suvival and migration towards an ischemic stimulus *in vivo*. 

## Results

### Survival and migration characteristics of locally injected BMSC

**Figure 1:**
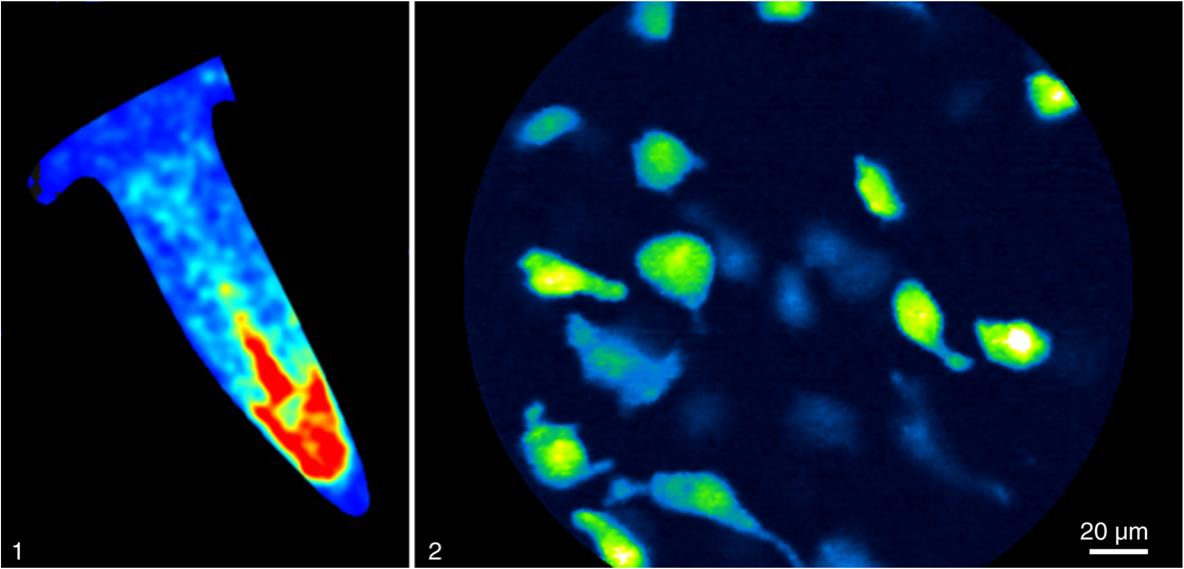
**Baseline expression of reporter genes before BMSC cell injection.** 1: Luciferase expression of BMSC measured with bioluminescence imaging. 2: *In vivo* endomicroscopy images of eGFP expressing BMSC before injection.

**Figure 2:**
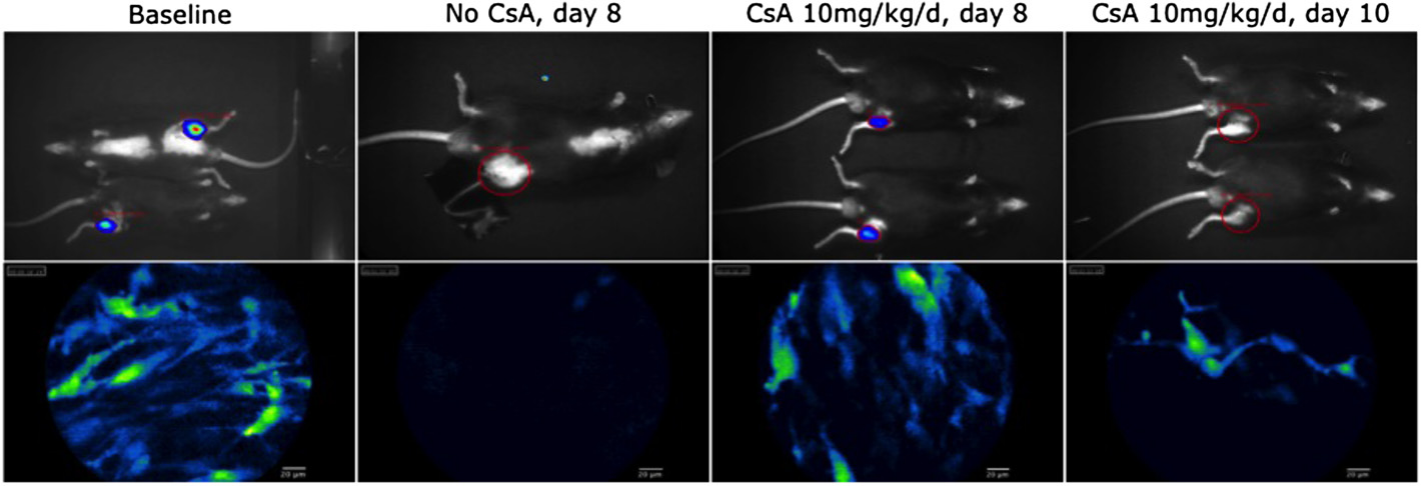
**Survival of IM injected BMSC visualized with bioluminescence (upper row) versus confocal endomicroscopy (lower row).** Immediately after IM injection a cell infiltrate was seen at the injection site. In the absence of immunosuppression, in most animals no residual cells were detectable beyond 8 days after cell transplantation. BMSC survival in mice treated with CsA was only temporarily prolonged. At day 8 post-transplantation some residual, viable cells were still observed, but the signal weakened rapidly during the second week after transplantation. No cell infiltrate was detected beyond 10 days after transplantation.

To study the *in vivo* survival and migration characteristics of IM injected BMSC, cells were transplanted into the calf muscles 24 h after induction of hindlimb ischemia. Prior to injection, eGFP fluorescence intensity and Luciferase activity were checked by confocal endomicroscopy and bioluminescence imaging methods, respectively (Figure 1). Mean (±SD) cell diameter was 13.4μm (±3.5). The biology of the cell transplant was investigated during a 3-week follow-up period using both *in vivo* imaging techniques. The high optical resolution (micrometer scale) of confocal endomicroscopy enabled us to perform a detailed study of the survival and differentiation characteristics of single cells in the BMSC transplant and to monitor BMSC homing towards the ischemic site. BMSC could be seen as a sharp delineated tissue infiltrate at baseline. The transplanted cells developed a spindle-shaped pattern with cell branches within a few days. The transplant appeared to be stable throughout the first week but then displayed a rapid and steep drop in BMSC number and density during the second week. As a result of the rejection of the BMSC transplant, cellular morphology became more heterogeneous in both size and appearance, less densely organized and more fragmented. BLI signals were in accordance with endomicroscopic findings (Figure 2). Signal intensity and position of the cell transplant remained fairly stable in the first week after transplantation. Hereafter, BLI signals rapidly faded to background levels in all animals during the second week after transplantation. Using BLI to discern active homing of the cell transplant towards the ischemic site (which is somewhat difficult because of the low optical resolution (± 1mm) of the BLI technique), we did not observe much change in the position of the cell transplant over time, nor was there evidence for active homing towards the ischemic site. To study the homing capacity of individual BMSC, we applied confocal endomicroscopy to the site of tissue ischemia. Here, the day after transplantation, only one eGFP positive cell could be found in the ischemic muscular region in only one transplanted animal. However, at all later time points, confocal endomicroscopy did not demonstrate homing of eGFP^+^ BMSC towards the area surrounding the ligation site Table 1.

**Table 1:** Comparison of detection techniques

	**Bioluminescence imaging**	**Confocal endomicroscopy**
**Strength**	Non-invasive	High spatial resolution
	Low background signals	Single cell imaging
	Highly specific for Luciferase activity	Cell morphology studies
**Weakness**	Low spatial resolution	Invasive procedure
	Low cell numbers not detectable	Requires direct probe contact
	Limited tissue penetration	Autofluorescence reduces specificity
	**Superior specificity**	**Superior spatial resolution**

### Ciclosporin immune suppression cannot sustain prolonged BMSC survival

**Figure 3:**
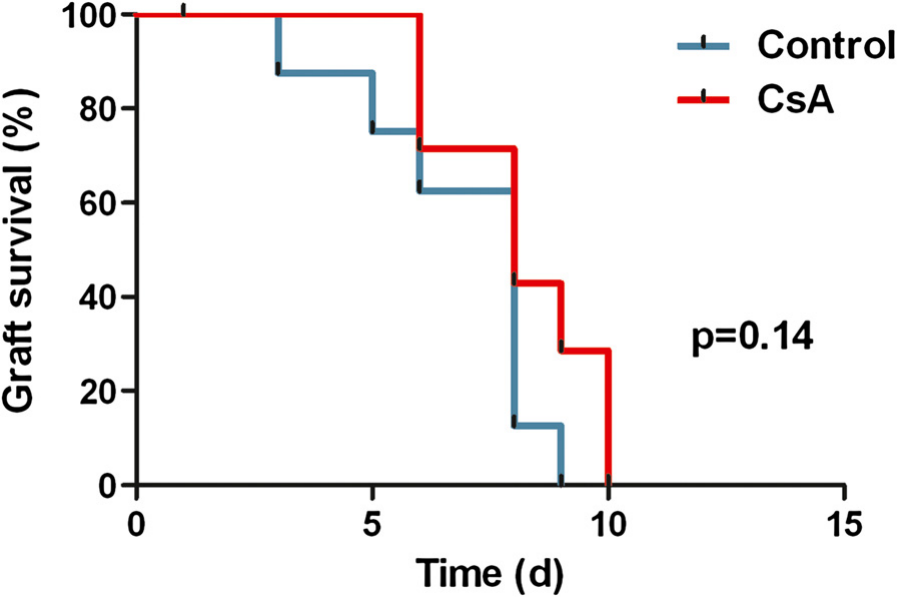
**BMSC transplant survival evidenced by BLI**/**endomicroscopy in IM injected animals treated with or without CsA.**

To study whether BMSC rejection could be delayed, we studied the rejection process of IM injected BMSC in animals treated with or without daily IP injections of CsA (n=8 for both groups). Using BLI and confocal endomicroscopy, we were able to show that this immunosuppressive regimen only marginally prolonged BMSC transplant survival, a difference that was not statistically significant (p=0.14) (Figure 3). To exclude the possibility of underimmunosuppression, a higher dose of CsA (30mg/kg/d instead of 10mg/kg/d) was used in about half of the animals. No apparent difference in BMSC transplant survival was observed between mice treated with either high- or low-dose immunosuppression.

### Intrapulmonary cell trapping of BMSC

**Figure 4:**
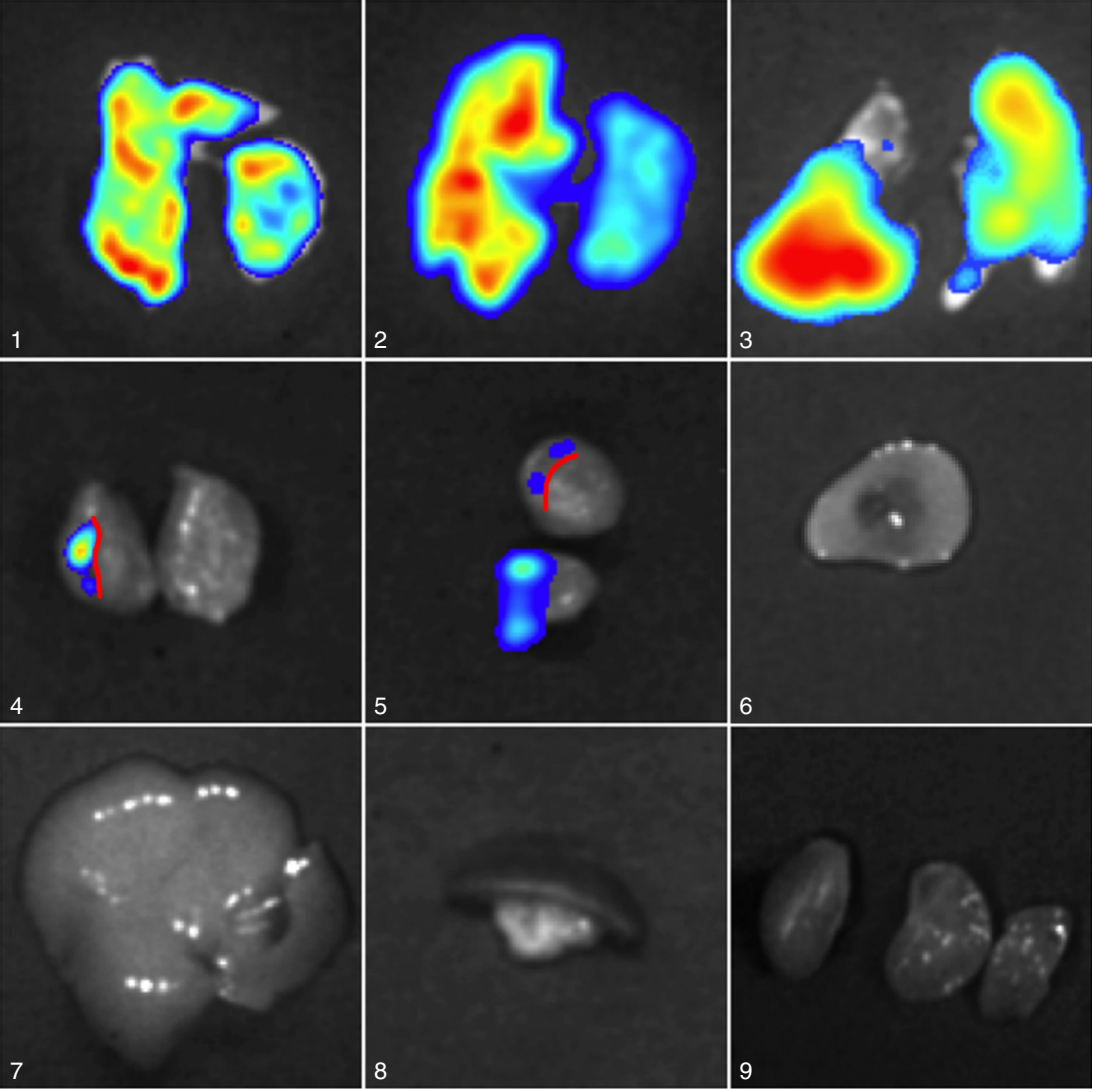
**BLI imaging of dissection specimens 30 min after CV injection of BMSC.** 1–5: Bioluminescent BMSC are present throughout the entire lung (3 independent experiments) and in the right heart ventricle (longitudinal (4) and cross-section (5), red bar depicts the interventricular septum). Note that the left ventricle is devoid of luminescent cells. 6–9: No signals are observed in the blood (6), or in other organs (liver (7); spleen (8); kidneys (9)).

**Figure 5:**
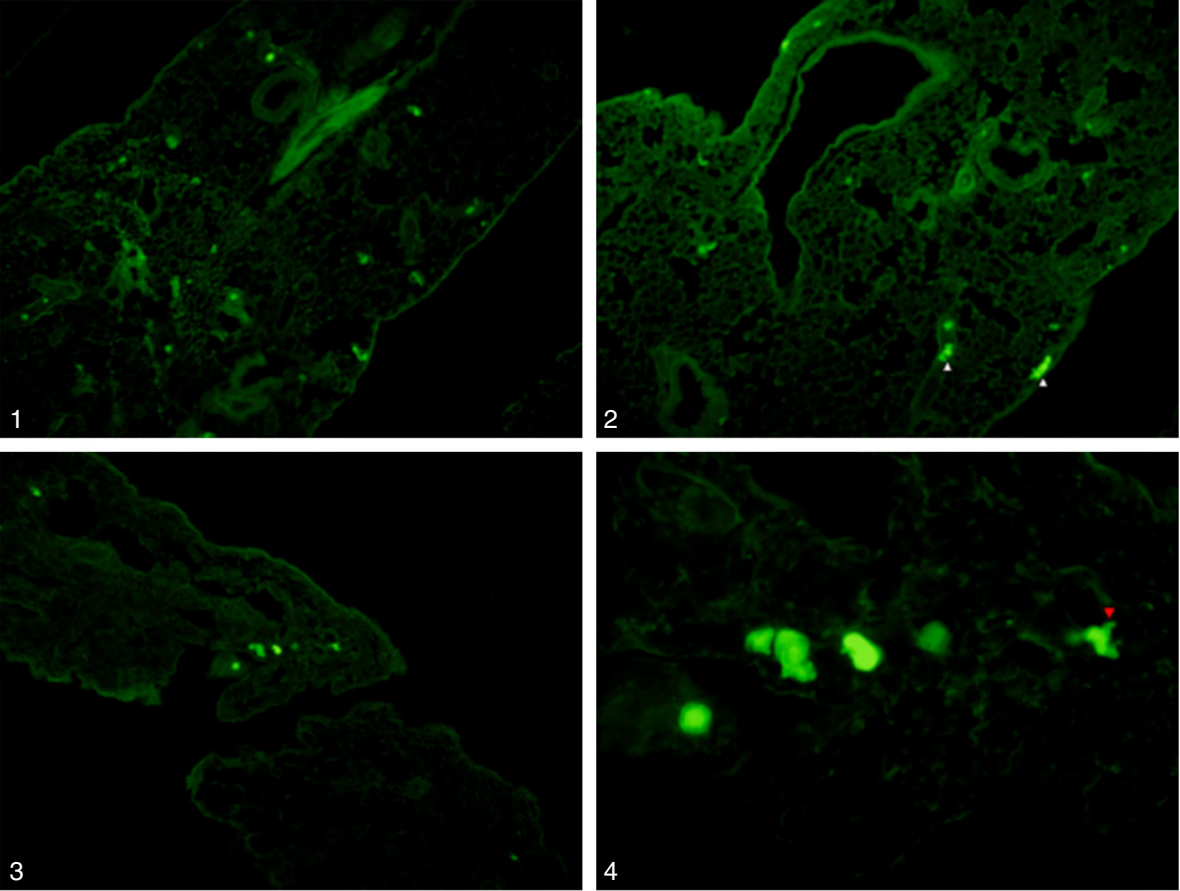
**Lung entrapment of BMSC.** 1–3: Fluorescence microscopic images of lung slices show eGFP^+^ BMSC as microemboli throughout the entire lung. Cells and cell aggregates can be seen in vascular structures (10×). 4: Higher magnification of image 3. Intravascular cell entrapment and diapedesis into the lung parenchyma (red arrowhead) of CV injected BMSC (40×).

CV injection of BMSC by right heart cannulation resulted in respiratory distress and led to respiratory failure and death within minutes (5/5). Confocal endomicroscopy on resection specimens revealed trapped eGFP^+^ BMSC in both lungs, the right ventricle and the right atrium. Intrapulmonary cell trapping was confirmed by BLI imaging 30 min after injection of D-luciferin pre-incubated BMSC. Bioluminescence signals were clearly visible in both lungs and were restricted to the right side of the heart. No BLI signal could be detected in the left atrium or the left ventricle, nor in tissue specimens of liver, kidney, spleen or blood (Figure 4). Intrapulmonary cell trapping after CV BMSC injection was further evident on fluorescence microscopic images of formalin-fixed, paraffin-embedded lung slices. eGFP^+^ cells were seen in small vessels or capillaries throughout the entire lung (Figure 5). Some cells appeared as round cells, while others displayed cell processes and seemed to evade the pulmonary circulation and enter into the lung parenchyma.

### Minimal evidence for BMSC homing after systemic transplantation

**Figure 6:**
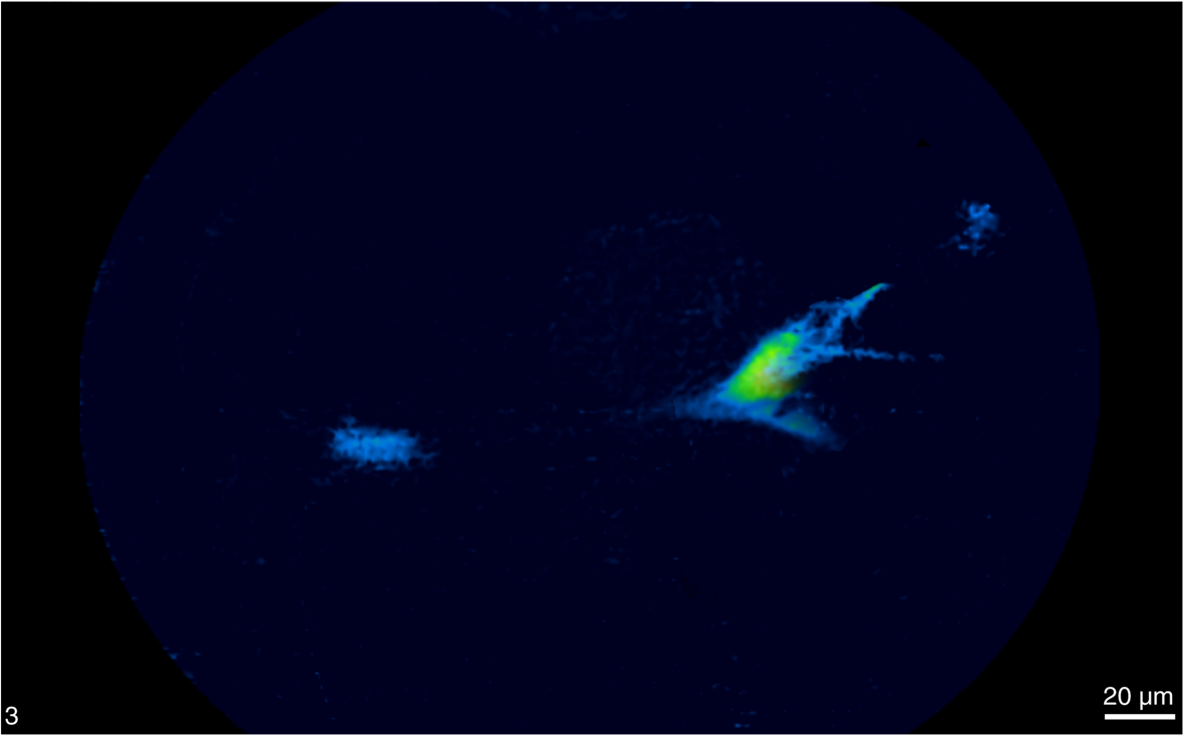
**Ischemia**-**directed homing of BMSC after intra**-**arterial injection.** Confocal endomicroscopic image of three BMSC at the ischemic site. Of note are the typical BMSC cell size, cellular morphology and eGFP^+^ fluorescence indicating living cells.

To study the process of ischemia-directed cell homing, BMSC were injected directly into the arterial circulation (n=5) by direct injection into the left heart ventricle, as such bypassing the pulmonary circulation. This procedure proved to be feasible and safe. During a two-week follow-up period, neither BLI nor confocal endomicroscopy demonstrated any convincing sign of active migration of BMSC towards the ischemic site in an immunocompetent host (n=2). However, in one out of three animals treated with CsA (10mg/kg/d), a cluster of three BMSC was detected by confocal endomicroscopy in the same microscopic plain of the ischemic hindlimb (Figure 6, video sequence provided as Additional file 1). Taking into account their typical morphology, size and fluorescence intensity, we feel confident that these cells are indeed living BMSC. Whether these cells actively migrated to the ischemic site or whether their presence was merely a chance phenomenon due to passive cell entrapment, is impossible to deduce from this limited amount of study data. This small number of detected cells, together with the scarce evidence for BMSC homing in IM transplantation experiments, led us to conclude that active BMSC homing is a rare event.

## Discussion and conclusions

In this study we investigated BMSC survival and ischemia-directed homing using two *in vivo* reporter gene expression imaging techniques, BLI and confocal endomicroscopy. Endomicroscopy has superior sensitivity in visualizing transplants at low cellular concentrations or with fading tracer signals. Its high optical resolution enables the study of cellular morphology, delineation of the cell infiltrate as well as homing capacity of individual cells. Drawbacks of the technique are its invasive nature requiring direct tissue contact with limited deep tissue penetration. Autofluorescence did occur at times but the chance of false positive findings in our study was rather limited because of the typical cell size and morphology of BMSC. BLI, on the other hand, is a non-invasive and hence more practical technique to use in follow-up studies of transplant survival, but it lacks the high optical resolution of endomicroscopy. It also has superior specificity for detecting Luc^+^ cells. However, BLI is unable to detect single cells or transplants with low cell concentration or limited cell numbers. Nevertheless, a combination of the high specificity of BLI joined with the high optical resolution of confocal endomicroscopy, overcomes several constraints of either technique alone and enables to study the biology of BMSC survival and homing with more precision and accuracy.

Studying cell homing using reporter genes has several advantages over conventional cell tracking agents, such as cell labeling dyes, microparticles, radioisotopes or magnetic resonance imaging (MRI) contrast agents. An important drawback of these conventional methods is that the agent used can become decoupled from the original cell transplant and/or ingested by other cell types, such as macrophages. Macrophage ingestion is a major confounder in transplantation studies because many cells of donor origin die shortly after cell transplantation, triggering a local immune reaction with macrophage infiltration [7].

Because reporter genes are integrated into the cell genome, cell tracking studies using reporter gene expression is much less likely to yield false positive results: the expression of the reporter gene is limited to viable cells of the original cell transplant and is preserved in successive cell divisions. When cells do not survive or are ingested by other cells, the signal diminishs over time. However, since the reporter gene is integrated in the genomic DNA, cell proliferation will increase rather than decrease signal intensity [4], as such preventing dilution of the original labeling signal.

Despite these benefits, the use of reporter genes also entails a number of caveats. A first problem could arise from the induction of immune responses directed against non-host alloantigens expressed on the cell surface of the transfected cells that express non-self proteins, such as Luc or eGFP. However, Bergwerf et al. [8] demonstrated that the immune reactive cytotoxic T-cell response following BMSC transplantation was not specific for the non-self eGFP or Luc genes. These authors suggested that the observed T-cell mediated transplant rejection could be due to the presence of xenogeneic constituents of the growth medium, such as fetal calf and horse serum, which render the cultured cells more immunogeneic. Another concern is gene silencing, which is a mechanistically very complex phenomenon that can vary with specific experimental conditions due to a site-dependent adaptation of the expression of the reporter gene *in vivo*[9].

We demonstrated in our hindlimb ischemia mouse model that cell transplant survival after IM injection was limited in time and that CsA immunosuppression alone was not sufficient to maintain long-term survival of the allogenic BMSC graft. Our data are in line with the BLI results of Swijnenburg et al [10]. In this study, immunosuppression with mycophenolate mofetil, sirolimus or tacrolimus, both in monotherapy or in combination, failed to induce long-term survival of embryonic stem cell xenografts. On the other hand, Swanger et al. [11] showed that, at least in the central nervous system, high dose CsA significantly increased allogeneic BMSC graft survival compared to standard dose CsA, indicating that higher doses of CsA could be sufficient to maintain long-term survival of allogeneic BMSC at immunologically privileged sites. Furthermore, evidence from BLI experiments from Zangi et al. [12] about survival and rejection of IV or intraperitoneally injected allogeneic MSC transplants, indeed showed that, at least in mice, survival of a systemically injected MSC transplant is limited in time. Moreover, it was demonstrated that even IM injection of BMSC in immune competent syngeneic mice did not result in permanent graft survival [8].

We used a hindlimb ischemia model for the study of ischemia-directed homing firstly for reasons of good accessibility of the tissues of interest for the optical probe of the confocal endomicroscope. Therefore, *in vivo* endomicroscopy would be less suitable to study homing towards deeper anatomical structures or to moving organs, such as the heart. Secondly, hindlimb ischemia mouse models have already been validated extensively as experimental set-up for the study of ischemia-directed MSC homing. A better understanding of survival and homing characteristics of MSC in this experimental model is of particular relevance for the early-phase clinical trials on the use of human MSC for the treatment of critical limb ischemia that are currently ongoing [13].

We caution against CV injection of large amounts of BMSC because this leads to massive pulmonary infarction. Gao and co-workers already reported that systemic injection of MSC initially results in retention in the lungs, after which a gradual redistribution could be seen to the liver, bone marrow and other organs. These authors showed that the pulmonary retention could be reduced using the vasodilator sodium nitroprusside [14]. This finding, i.e. initial intrapulmonary cell trapping with gradual tissue redistribution, was confirmed by Daldrup-Link et al. [15], who used MRI imaging, and by *in vivo* BLI studies by Schrepfer et al. [16]. In the latter study, episodes of tachypnea, apnea and hemodynamic alterations, characteristic of pulmonary embolism, were reported when MSC were directly infused into the inferior vena cava. Pulmonary entrapment was also evident in a myocardial infarction study with BrdU-labeled MSC [6]. When the lung circulation was bypassed through direct intracavitary administration of MSC, significantly more cells were observed within the infarction zone within the first hours after transplantation. In a later report by the same authors, long-term engraftment of the infused MSC, as initially suggested on the MRI images, could not be verified with Y chromosome gene detection. Further analysis revealed that the positive MRI signals originated from cardiac resident macrophages that had ingested the MRI contrast particles. Intriguingly, although cells of donor origin were absent at 4 weeks after infusion, ventricular function was still improved in the MSC-treated animals [17]. The authors attributed this phenomenon to possible paracrine effects. Another tissue distribution study in the clinical setting of myocardial infarction, showed initial lung uptake of intravenous (IV) injected ^18^F-FDG-labeled bone marrow-derived cells, followed by a gradual redistribution to the reticulo-endothelial system (spleen, liver, bone marrow) [18]. Stem cell homing towards the infarction site, however, could not be demonstrated. Evidence for the pulmonary entrapment of stem cells was also reported after IV injection of neural stem cells [19], leading to massive pulmonary inflammation and apoptosis. Together, these studies indicate that the lung might act as a barrier for the passage of different stem cell subtypes [20], especially for those that are relatively large and express abundant adhesion molecules [21]. Our study adds further evidence to the finding of intrapulmonary cell trapping after CV injection of BMSC and questions the safety of this route for BMSC transplantation.

As indicated by our results, direct intracavitary injection into the left ventricle is a feasible approach to circumvent this pulmonary “first-pass” effect, but it is rather impractical to use in small animal experiments. However, direct IA administration of BMSC in the arterial circulation, e.g. by intracoronary injection after myocardial infarction, thereby avoiding the lung circulation, would in our opinion prove to be the method of choice for BMSC cell application in large animal and human clinical trials. Furthermore, considering that active ischemia-directed BMSC homing of unstimulated BMSC was a rare biological phenomenon in our studies, this also favours direct IA injection of BMSC into the vasculature of the tissue of interest. Alternatively, the homing process could potentially be improved with use of stimulated rather than naive BMSC. BMSCs can easily be ex vivo manipulated by pretreatment, preconditioning and genetic modification, which was reported to increase their therapeutic benefit [22]. For instance, hypoxic proconditioning has been shown to stimulate the induction of several chemokine receptors, such as CXCR4 and CXCR7. This led to improved migration and recruitment of BMSC into ischemic tissues and stimulated secretion of proangiogenic and mitogenic factors improving therapeutic potential of the transplanted BMSC [23].

As a limitation of our work, we are aware that we could have underestimated the BMSC homing process because of the specific limitations of our detection techniques and experimental set-up. However, by combining the advantages of BLI with endomicroscopy, we feel confident about our results. However, it is possible that we underestimated the homing potential of BMSC, because of the limited graft survival time or the use of naive rather than stimulated or manipulated BMSC. Also, the use of CsA could have interfered with BMSC kinetics or altered the BMSC microenvironment, leading to an impaired homing respons. Transgenic immunodeficiency models, rather than immunosuppression with pharmacological agents, would be an interesting alternative to use in future studies on BMSC survival and homing. Finally, increasing the number of observations in each group would further strengthen our statements.

In summary, in this study a combination of BLI and confocal endomicroscopy was applied for dual reporter gene-expression imaging of BMSC survival and homing in a clinically relevant mouse model of peripheral tissue ischemia. We report that allogeneic BMSC graft survival time was limited in the ischemic muscle and that CsA immunosuppression alone was not able to sustain long-term survival of the allograft. We did not find abundant evidence for ischemia-directed BMSC homing as a common biological process and caution against the direct CV injection of large amounts of BMSC.

## Methods

### Animals

Homozygous ROSA26-L-S-L-Luciferase transgenic mice (FVB background) were purchased from Jackson Laboratories (strain 005125) and further bred in the specific pathogen-free animal facility of the University of Antwerp. Male offspring was used for BMSC culture. Male C57Bl/6 mice (Charles River) (n=26) were used as acceptors. Animals were kept in a normal day-night cycle (12/12) with free access to food and water. National and European principles of laboratory animal care were followed. All animal experimental procedures were approved by the Animal Care and Use Committee of the University of Antwerp (Permit Number: 2008–03).

### Reporter gene-expressing bone marrow-derived stromal cells (BMSC)

BMSC genetically engineered with the Luciferase (Luc) and enhanced Green Fluorescent Protein (eGFP) reporter proteins were cultured and phenotypically characterized as previously described by Bergwerf et al. [8]. eGFP^+^/Luc^+^ BMSC were cultured in “complete expansion medium” (CEM) consisting of Iscove’s modified Dulbecco’s medium (Cambrex) supplemented with 8% fetal bovine serum (HyClone Laboratories), 8% horse serum (Invitrogen), 100 U/mL penicillin (Invitrogen), 100 mg/mL streptomycin (Invitrogen), 0.25 mg/mL amphotericin B (Invitrogen). CEM was supplemented with 1 μg/mL Puromycine (Invivogen) to induce eGFP expression (eGFP-IRES-Pac cassette). Cultures were harvested twice a week using trypsin-EDTA (Invitrogen) and passaged at a 1:3 ratio in 15ml CEM in T75 culture flasks. Cells of passage number 5 to 20 were used in the experiments. Before transplantation, eGFP^+^/Luc^+^ BMSC were washed twice in sterile PBS solution and resuspended at a final concentration of 5.10^6^ BMSC/ml NaCl 0,9%. Cell preparations were kept on ice until cell injection.

### Hindlimb ischemia mouse model and cell transplantation

Hindlimb ischemia was induced by ligation of the left femoral artery (LFA). In brief, 3- to 5-month-old male C57BL/6 mice were anesthetized (demetomidine 0.4ml/kg and ketamine 37.5mg/kg, intraperitoneally). The LFA was ligated as close as possible to the left inguinal ligament using 7–0 polypropylene sutures (Prolene®, Ethicon, Johnson & Johnson). Subsequently, the operation wound was closed and animals were placed under a heat source until full recovery. After 24h, mice were re-anesthetized and transplanted with 500.10^3^ eGFP^+^/Luc^+^ BMSC dissolved in 0.1ml NaCl 0.9%. The transplant route was either central venously (CV) by right heart cannulation (n=5), intra-arterial (IA) by left ventricular cannulation (n=5) or intramuscular (IM) directly into the calf muscle (n=16). For right and left heart cannulation, animals were intubated (23G catheter) and mechanically ventilated (10μl/g, 180/min, 2cm H_2_O positive end-expiratory pressure) and a small parasternal thoracotomy was performed. Systemic injection was performed slowly at a rate of 100μl/min. To study the effects of the presence or absence of immunosuppressive therapy on immune rejection of the cell transplants, animals were treated with (n=13) or without (n=13) daily intraperitoneal injection of CsA. Two dosage regimens were compared: 10mg/kg/d (n=6) versus 30mg/kg/d (n=7).

### Confocal endomicroscopy

Homing of eGFP^+^/Luc^+^ BMSC was visualized *in vivo* with the Cellvizio® LAB system (Mauna Kea Technologies) equipped with a 488nm excitation laser. Cellvizio® LAB is a high-speed fluorescence microscope that permits real-time endoscopic fluorescence imaging through high-resolution fiber bundle micro-optics (UltraMini0 microprobe: tip diameter 2.6mm, lateral resolution 1.4μm, field of view 240μm, working distance 60μm). In each mouse, cell homing was visualized 3 times per week, for a time period of 3 weeks. In brief, mice were anesthetized (demetomidine 0.4ml/kg and ketamine 37.5mg/kg, intraperitoneally). A small skin incision was made in the inguinal region, the microprobe was advanced towards the ligation site and the underlying muscular tissues were scanned for the presence of fluorescent cells. A second incision at the level of the calf muscles enabled us to study the morphology, survival and homing characteristics of the IM injected cell infiltrate distal to the ligation site. Tissues were imaged for at least 5 min with continuous video recording. The contralateral hindlimb served as a negative control to discriminate eGFP^+^ cell homing from autofluorescence background signals. After the procedure, the operation wounds were closed using 7–0 polypropylene sutures (Prolene®, Ethicon, Johnson & Johnson).

### *In vivo* bioluminescence imaging (BLI)

In all transplanted mice, homing of viable eGFP^+^/Luc^+^ BMSC was analyzed with a non-invasive *in vivo* photon-imager system (Biospace), three times weekly for 3 weeks (or less in case BLI signals consistently disappeared earlier). Mice were anesthetized (demetomidine 0.4ml/kg and ketamine 37.5mg/kg, intraperitoneally) and intraperitoneally injected with 100μl D-luciferin (30mg/ml, dissolved in PBS) with a 29G needle. After 10 min, bioluminescence signals were recorded for at least 10 min. Bioluminescence recordings were layered onto a visual photographic image to create composite bioluminescence - photographic images. The data were analysed with Photovision software. To study the phenomenon of intrapulmonary cell trapping, CV injected BMSC were pre-incubated with D-Luciferin. Bioluminescence images of the dissected lungs, heart, spleen, kidneys, liver and blood were obtained postmortem.

### Histology – fluorescence microscopy

Tissue specimens were fixed in 4% paraformaldehyde for 2 h at room temperature, washed 3 times with PBS and stored overnight in 70% ethanol. Tissues were paraffin-embedded, sliced into 5μm thick tissue sections and analyzed for eGFP^+^ cells using a Zeiss Axiophot fluorescence microscope equipped with an DP70 digital camera system (Olympus). Images were analyzed in Cell^P^ Imaging Software version 3.4 (Olympus).

### Statistical analysis

Logrank test was used for curve comparison of survival data. A p-value of <0.05 was considered to be statistically significant.

## Abbreviations

BLI: Bioluminescence imaging; BMSC: Bone marrow stromal cell; CEM: Complete expansion medium; CsA: Ciclosporin A; CV: Central venous; eGFP: Enhanced green fluorescent protein; IA: Intra-arterial; IM: Intramuscular; IV: Intravenous; LFA: Left femoral artery; MRI: Magnetic resonance imaging; MSC: Mesenchymal stem cell; SD: Standard deviation.

## Competing interests

The authors declare that they have no competing interests.

## Authors’ contributions

BRE carried out the animal studies, bioluminenscence, confocal endomicroscopy, histology, study design and drafted the manuscript. IB, NVD and PP provided BMSC, participated in study design and drafted the manuscript. AVDL provided BLI and confocal endomicroscopy and helped to draft the manuscript. JPT and CJV conceived of the study, participated in its design and coordination and helped to draft the manuscript. All authors read and approved the final manuscript.

## Supplementary Material

Additional file 1 BMSC cluster. Click here for file
